# Insight into the Interaction Mechanism of Pseudorabies Virus Infection

**DOI:** 10.3390/biology13121013

**Published:** 2024-12-04

**Authors:** Xiaoyong Chen, Ziding Yu

**Affiliations:** 1 Xingzhi College, Zhejiang Normal University, Lanxi 321100, China; 2 College of Veterinary Medicine, China Agricultural University, Beijing 100193, China

**Keywords:** pseudorabies virus, Aujeszky’s disease, virus–host interaction, viral pathogenesis

## Abstract

The pseudorabies virus (PRV), also known as suid alphaherpesvirus 1 (SuAHV-1), has garnered significant attention due to its broad host range and the economic losses it incurs in the swine industry. This review aims to provide a comprehensive understanding of the intricate virus–host interactions during PRV infection, focusing on the evasion strategies of the virus against the host responses. We also summarize the mechanisms by which PRV manipulates the host cell machinery to facilitate its replication and spread, while simultaneously evading detection and clearance by the immune system. Furthermore, we discuss the latest advancements, such as metabolic, autophagic, and apoptotic pathways in studying these interactions, highlighting the role of various cellular factors and pathways in elucidating virus–host dynamics. By integrating these insights, the article aims to provide a comprehensive overview of the molecular mechanisms underlying PRV pathogenesis and host response, paving the way for the development of novel therapeutic strategies against this virus.

## Simple Summary

The pseudorabies virus (PRV) remains a devastating pathogen in swine, significantly impacting animal health and causing substantial losses in the breeding industry. By summarizing the latest advancements, including innate immunity, metabolic pathways, autophagic processes, protein degradation systems, and others, this work underscores the pivotal role of various cellular factors and pathways in shaping the PRV–host dynamic. Additionally, the work highlights viral sophisticated escape mechanisms that enable it to evade host defenses for self-replication. We hope that this work can provide a profound understanding of PRV–host interactions, laying the groundwork for the development of innovative therapeutic strategies to combat this virus.

## 1. Introduction

The history of the pseudorabies virus (PRV) spans several decades, tracing back to its initial discovery in the early 19th century [1]. Initially, the virus was often misidentified as rabies due to the similar neurological symptoms exhibited by infected animals. However, subsequent research revealed distinct differences between the two viruses, resulting in the establishment of PRV as a separate entity. Throughout the 19th and early 20th centuries, PRV was primarily studied in Europe, where it was recognized as a serious threat to the swine industry [2]. Early reports described outbreaks among pigs, characterized by severe neurological signs, fever, and high mortality rates. These outbreaks caused significant economic losses and raised concerns among farmers and veterinarians [3]. In the mid-20th century, the virus gained increasing attention from the scientific community, leading to more extensive research efforts [4]. Scientists began to characterize the virus’ physical properties, genetic structure, and replication cycle. This period marked a significant milestone in understanding PRV pathogenesis and transmission dynamics. Today, PRV remains a relevant pathogen in the animal industry, particularly in regions where vaccination programs are not widely implemented. Ongoing research efforts continue to explore new ways to prevent, control, and treat PRV infections, aiming to further reduce its impact on animal health and production.

**Figure 1:**
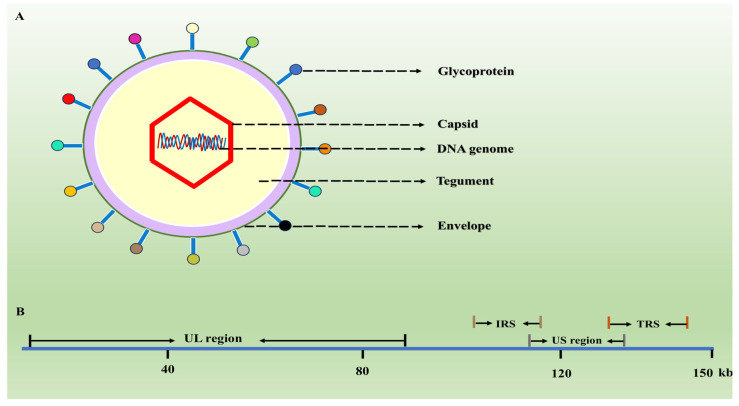
The structure of PRV virion and genome. (**A**) PRV virions consist of five components, encompassing a double-stranded DNA genome, an icosahedral protein capsid, a tegument protein layer, an envelope, and surface glycoproteins. (**B**) The PRV genome comprises the unique long region (UL) and unique short region (US), bordered by the internal repeat sequence (IRS) and terminal repeat sequence (TRS).

PRV, a member of the *Herpesviridae* family, exhibits distinct virus particle features. Its virions, visible under electron microscopy, possess a complex and unique structure, with a typical diameter ranging from 150 to 200 nanometers [5]. These virions are characterized by an icosahedral capsid composed of multiple protein subunits, enclosing a double-stranded DNA genome [6]. The genome is organized into unique long (UL) and unique short (US) regions, flanked by the internal repeat sequences (IRS) and terminal repeat sequences (TRS), encoding over 70 proteins essential for replication, virulence, and immune evasion (Figure 1) [7]. These proteins can be grouped into structural, enzymatic, regulatory, and host–virus interaction categories [8]. Structurally, PRV encodes several proteins that form the virion capsid, tegument, and envelope. The capsid proteins, such as the major capsid protein and the triplex proteins, assemble to form the icosahedral shell that encapsulates the genome of the virus [9]. The tegument proteins, located between the capsid and the envelope, perform various functions during virus entry, replication, and egress. The glycoproteins mediate virus attachment to host cells and fusion to the cellular membranes, enabling virus entry into the cell [10]. In terms of enzymatic functions, PRV encodes proteins such as thymidine kinase and dUTPase, which are involved in nucleic acid metabolism and play crucial roles in viral replication [11,12]. Additionally, PRV expresses several regulatory proteins that modulate gene expression at different stages of infection, ensuring the timely production of viral proteins and efficient replication [13,14]. Furthermore, PRV possesses proteins that interact with the host immune system, enabling the virus to evade immune detection and clearance [15]. These proteins include viral homologs of cellular cytokines and chemokines, which can mimic or antagonize their host counterparts, and proteins that interfere with host signaling pathways involved in immune responses.

PRV poses significant threats to both animal health and the agriculture industry. It is highly contagious, affecting a wide range of mammal species, including pigs, cattle, dogs, cats, and even wildlife such as foxes and raccoons [16]. One of the primary dangers of PRV is its ability to cause severe neurological damage. Upon infection, the virus initially replicates in epithelial tissues, subsequently invading the peripheral nervous system via sensory nerve endings. This invasion is followed by the retrograde axonal transport to the central nervous system (CNS), where PRV can induce a range of pathological changes, including neuronal death, inflammation, and alterations in neurotransmitter systems. The resultant clinical manifestations often include severe neurological symptoms such as ataxia, paralysis, and even death, highlighting the profound impact of PRV infection on neuronal function and integrity [8,17]. In addition to its neurological effects, PRV could lead to respiratory distress, fever, anorexia, and eventually death in severe cases. This virus spreads rapidly through bodily fluids and direct contact, making it difficult to eliminate once an outbreak occurs [18]. Outbreaks among livestock, particularly pigs, can result in substantial losses for farmers due to decreased production, increased mortality rates, and the need for costly veterinary interventions. Furthermore, the spread of PRV could disrupt trade and export markets, affecting the global economy. Beyond its direct effects on animals and agriculture, PRV also poses a threat to human health [19]. Although humans are not typically susceptible to developing the disease, they can be infected by animals and manifest clinical symptoms, such as fever, headache, sweating, encephalitis and endophthalmitis [20]. This risk is heightened in occupations that involve close contact with animals, such as farming, veterinary medicine, and wildlife conservation.

PRV infections have been reported in numerous countries, with varying degrees of impact on their respective swine populations [21]. In Europe, several countries such as Germany, the Netherlands, Denmark, and Sweden, have successfully eliminated pseudorabies from their pig populations through rigorous vaccination programs and biosecurity measures [22]. However, the threat of re-emergence remains, particularly due to the potential introduction of the virus from unvaccinated or inadequately controlled areas. Asian countries, such as Japan, Vietnam, and Thailand, also reported cases of pseudorabies, highlighting the need for continued efforts in disease control and prevention [23,24,25]. The use of genetically engineered vaccines tailored to address the emerging variants is being explored as a potential solution. Notably, the virus’ ability to mutate and adapt has led to the emergence of different strains, complicating efforts to contain its spread [26]. China, in particular, has experienced a resurgence of the disease since 2011, with new variants of the virus emerging. These variants have been found to be more virulent, causing higher mortality rates and posing a threat to the country’s pig industry. Despite widespread vaccination, many pig farms, especially those with inadequate biosecurity measures, continue to struggle with pseudorabies outbreaks. Additionally, the global trade of live animals and animal products has facilitated the transboundary movement of PRV, leading to its establishment in new geographical areas in China [27]. Over the years, China has implemented several control measures, including vaccination programs and biosecurity enhancements, to mitigate the impact of PRV. However, outbreaks continue to occur, particularly in areas with inadequate vaccination coverage or biosecurity lapses. The Chinese government and research institutions have been proactive in monitoring PRV evolution and responding to outbreaks [28]. Vaccination remains the cornerstone of PRV control and vaccination programs have been widely implemented in China, with various vaccines available to protect swine against the virus [29]. It is crucial to ensure high vaccination coverage, particularly in high-risk areas, and to update vaccination strategies in response to emerging virus strains. Enhancing biosecurity measures on farms is vital to prevent the introduction and spread of PRV. This includes strict access controls, proper disinfection of facilities, and segregation of sick animals. Farmers should also be educated on the importance of biosecurity and how to implement it effectively. Continuous surveillance and monitoring are essential for the early detection of and rapid response to PRV outbreaks, involving regular testing of swine populations, tracking virus transmission patterns, and monitoring for changes in virus strains or virulence.

Additionally, many countries continue to maintain strict import regulations for pigs and pork products, conducting regular surveillance in wildlife populations that may act as reservoirs for the virus, and maintaining high levels of biosecurity on pig farms. Wild boars are particularly susceptible to PRV infection [30]. The virus can cause severe disease in this species, with symptoms similar to those seen in domestic pigs, including respiratory problems, neurological signs, and reproductive failure. Infected wild boars may experience a high mortality rate, especially if they are not vaccinated or if the virus strain is highly virulent. The virus can persist in wild boar populations, acting as a reservoir for infection [31]. This persistence can lead to sporadic outbreaks in domestic pig farms, particularly if biosecurity measures are not adequate. The movement of wild boars, either naturally or through human-mediated activities, such as translocation for hunting purposes, can spread the virus to new areas and increase the risk of disease transmission to domestic pigs [32]. However, the clinical significance of PRV infection in other species is generally low, and they are not considered major reservoirs for the virus. In some cases, PRV infection in non-pig species may result in mild or subclinical disease, with no overt symptoms. However, the potential for cross-species transmission and the role of these species as sentinels for PRV circulation in the environment require further investigation. The impact of PRV on wild boar populations can have broader ecological consequences. Wild boars play important roles in ecosystems as prey species for predators and as dispersers of seeds and nutrients [33]. A significant reduction in wild boar populations due to PRV infection could disrupt these ecological processes and affect the balance of predator–prey relationships. Furthermore, the presence of PRV in wild boar populations may influence the distribution and dynamics of other pathogens. For example, co-infection with PRV and other viruses or parasites could alter disease outcomes or transmission patterns, with potential implications for wildlife health and disease management.

PRV remains a significant threat to the swine industry and has the potential to infect humans. A comprehensive understanding of how hosts take up weapons to fight against PRV is essential to the pathogenic mechanism of this virus and to mitigate the impact of this virus. In this review, we discuss how hosts respond to PRV invasion, including utilizing innate immunity, autophagy, apoptosis, and modifications to clear PRV infection. In addition, we highlight how PRV antagonizes the host reaction by various pathways.

## 2. Interaction with Innate Signaling

**Figure 2:**
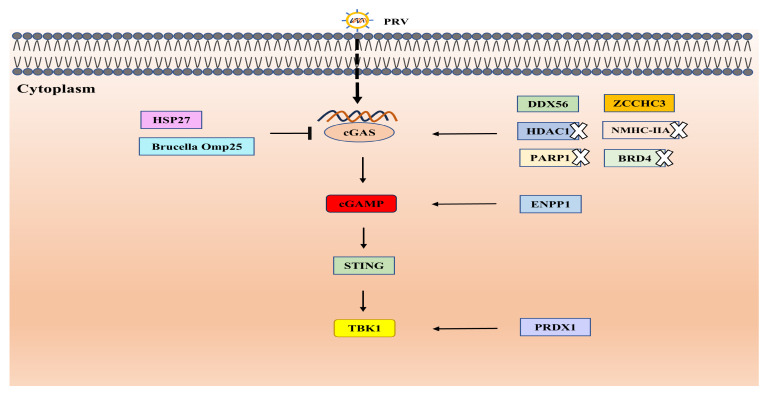
Factors involved in the regulation of cGAS–STING signaling during PRV infection. HSP27 and Brucella Omp 25 targets cGAS for degradation, while DDX56 and ZCCHC3 promote cGAS levels. Meanwhile, inhibition of HDAC1, PARP1, BRD4, or NMHC-IIA could enhance cGAS expression via inducing DNA damage or other pathways. ENPP1 positively regulates the homeostasis of cGAMP and PRDX1 interacts with TBK1.

It is widely known that the host innate immune response is triggered by recognizing viral components upon invasions [16]. PRV can be detected by several pattern recognition receptors (PRRs), among which cyclic guanosine monophosphate-adenosine monophosphate synthase (cGAS) holds the primary responsibility for its identification. cGAS senses the presence of PRV and disrupts its replication process by promoting the generation of cyclic GMP-AMP (cGAMP) and activating interferon-beta (IFN-β) signaling [34]. Additionally, ectonucleotide pyrophosphatase phosphodiesterase 1 (ENPP1) maintains the homeostasis of cGAMP, thereby regulating PRV replication [35]. DEAD-box proteins, which are ATP-dependent RNA helicases crucial for RNA metabolism and the host antiviral defense, also play a role. Notably, DEAD-box helicase 56 (DDX56), a conserved antiviral factor, impairs PRV replication by elevating cGAS expression and IFN levels [36]. Likewise, zinc finger CCHC-type containing protein 3 (ZCCHC3) has been found to augment cGAS levels and activate cGAS signaling, thus restricting PRV infection [16]. The inhibition of histone deacetylase 1 (HDAC1), poly (ADP-ribose) polymerase 1 (PARP1), non-muscle myosin heavy chain IIA (NMHC-IIA), or bromodomain protein 4 (BRD4) activates the cGAS-stimulator of IFN genes (STING) pathway, further impeding PRV replication [37,38,39,40]. Recent studies by Lv et al. reveal that peroxiredoxin 1 (PRDX1) hinders PRV infection by enhancing IFN production through its interaction with TANK-binding kinase 1 (TBK1) and IκB kinase ε (IKKε) [41]. Conversely, the heat shock protein 27 (HSP27) undermines the innate immune response to PRV invasion by targeting cGAS for proteasomal degradation, favoring viral replication [42]. Interestingly, the outer membrane protein (Omp) 25 from Brucella also targets cGAS for proteasomal degradation, disrupting IFN production, downstream antiviral genes, and ultimately inhibiting PRV replication [43]. These factors that are involved in the regulation of cGAS–STING signaling are shown in Figure 2.

IFN-stimulated genes (ISGs) play a crucial role in restricting virus replication and spread at multiple levels. They can interfere with viral entry by altering cell membrane properties, disrupt viral gene expression and replication by targeting viral nucleic acids or proteins, and inhibit virus assembly and egress by modifying the intracellular environment [44]. Several ISGs that specifically target and inhibit various stages of the virus life cycle have been identified. For instance, oligoadenylate synthetase-like (OASL) and ISG20 can hinder PRV replication by amplifying retinoic-acid-inducible gene I (RIG-I) signaling, as evidenced in studies [45,46]. Additionally, porcine myxovirus resistance 1 (Mx1) and Mx2 proteins impede PRV infection through their guanosine triphosphatase (GTPase) activity and the formation of stable oligomers [47]. It is intriguing to note that non-POU domain-containing octamer-binding protein (NONO), categorized as a Drosophila behavior/human splicing (DBHS) protein, counters PRV infection by bolstering IFN signaling [48]. Recently, cathelicidin B1 (CATH-B1) has emerged as a novel antiviral factor against PRV infection. This is supported by the elevated expression of IFN-β and ISGs facilitated by the Toll like receptor 4 (TLR4)/c-Jun N-terminal kinase (JNK)/IFN regulated factor 3 (IRF3) pathway [49]. Furthermore, the IFN-inducible transmembrane (IFITM) proteins constitute a family of small homologous proteins crucial for viral resistance [50]. It is indicated that their efficacy in restricting infections caused by various viruses, including the West Nile virus (WNV), dengue virus (DENV), and PRV. Specifically, PRV-induced IFITM1 expression is linked to the cGAS/STING pathway, and in turn, IFITM1 acts as a suppressor by impeding PRV entry [51]. Moreover, another member of this family, IFITM2, disrupts virus binding and entry through the cholesterol pathway [52].

**Figure 3:**
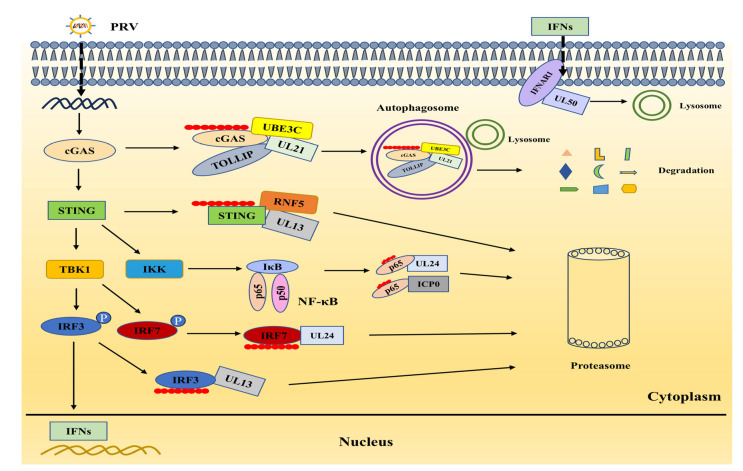
The viral proteins directly target the cGAS–STING axis for proteasomal or lysosomal degradation. PRV infection triggers cGAS–STING signaling in the host, which in turn blocks PRV replication. While several viral proteins directly interact with the factors in this pathway, they target these immune factors for degradation, thus antagonizing the host innate immune response. UL21 recruits E3 ligase to degrade cGAS and STING via autophagy-lysosomal pathways. UL50 targets IFNAR1 for lysosomal degradation. UL24, UL13, and ICP0 target various immune factors for proteasomal degradation.

Nevertheless, PRV has devised numerous strategies to counteract the host innate immune signaling, enabling successful infection (Figure 3). Recent studies have uncovered specific mechanisms employed by PRV. UL21 was found to engage the E3 ligase ubiquitin protein ligase E3C (UBE3C) to catalyze the K27-linked ubiquitination of cGAS at Lys384. Subsequently, this modified cGAS is recognized by the cargo receptor toll interacting protein (TOLLIP) and transported to the lysosome for degradation [53]. Kong and colleagues revealed another intriguing mechanism: PRV tegument protein UL13 suppresses STING-induced antiviral signaling by destabilizing STING. This process involves UL13 binding to the STING CDN domain, recruiting the E3 ligase RING-finger protein 5 (RNF5) to catalyze the STING’s ubiquitination and degradation. Notably, the absence of RNF5 enhances the host immune response against PRV [54]. Moreover, UL13 targets PRDX1, which interacts with TBK1 for degradation via the ubiquitin-proteosome pathway, further antagonizing antiviral effects [41]. The roles of other viral proteins, such as UL24, UL50, gE, and EP0, in countering the innate immune system have been comprehensively addressed in earlier publications [15,55]. The intricate relationship between PRV and antiviral factors illustrates a fascinating aspect of the ongoing evolutionary arms race between viruses and their hosts. Delving into the molecular mechanisms that underlie this interaction offers valuable insights into virus pathogenesis and host immune responses, potentially paving the way for innovative antiviral strategies.

## 3. Interaction with Metabolic Pathways

Viruses heavily depend on host metabolic pathways to achieve their replication. Thus, metabolic pathways could also be the target in regulating PRV replication. It was found that PRV infection altered metabolite levels, particularly in glycolysis, amino acid, and nucleotide metabolism pathways. Inhibiting glycolysis and the pentose phosphate pathway reduced viral replication, while inhibiting oxidative phosphorylation had minimal effect. Glutamine starvation decreased viral titers, but inhibiting glutaminase or adding 2-ketoglutarate did not alter PRV replication [56]. Moreover, Yao et al. comprehensively analyzed metabolite changes in porcine alveolar macrophage cells infected with different PRV strains: vaccine strain (Bartha K61), classical strain (EA), and variant strain (HNX). The infected cells showed significant metabolite differences compared to uninfected cells, with lipids and lipid-like molecules accounting for over 50% of the altered metabolites. The study reconstructed major lipid metabolic pathways involved in PRV infection, revealing both common and strain-specific metabolic changes. HNX infection caused unique metabolic alterations, potentially linked to its distinct biological characteristics and pathogenicity [57]. Recently, Liu et al. analyzed metabolic changes in PK-15 cells infected with two strains. PRV infection significantly alters many metabolites, especially those involved in lipid, purine, and pyrimidine metabolism, which may favor virus replication. Both strains show similar metabolic reprogramming, providing insights into PRV pathogenesis and antiviral strategies [58]. These above studies suggest that PRV infection reprograms PK-15 cell metabolism, with metabolic flux from glycolysis and glutamine metabolism to nucleotide biosynthesis being essential for enhancing viral replication.

Cholesterol, a lipid molecule found in cell membranes, plays a crucial role in various cellular processes, including the replication of certain viruses [59,60,61]. Statins hinder cellular cholesterol production. Treating SK cells with lovastatin before infection with PRV did not alter the production of viral proteins within the cell but decreased virus quantity. Removing cholesterol from the virus envelope also lowered virus levels, while adding external cholesterol reversed these effects. Lower cholesterol levels were found to reduce both the infectivity and stability of the newly produced virus [60]. Similarly, Song et al. found that U18666A, a cholesterol transport inhibitor, intervened with PRV infection. They further demonstrated that U18666A significantly reduces the production of infectious PRV particles, specifically suppressing their release. Pretreating target cells with U18666A reduces virus yield, while pretreating virions has no effect [62]. Another factor sphingomyelin (SM), found in the membrane of mammalian cells, is associated with cholesterol to form lipid raft domains. While treating cells with sphingomyelinase reduced cell surface sphingomyelin, it inhibited the entry of the PRV. Furthermore, inhibiting host acid sphingomyelinase hindered PRV entry but not herpes simplex virus 1 (HSV-1) entry. These findings indicate variations in alpha-herpesvirus entry requirements for cellular sphingomyelin and acid sphingomyelinase activity [63]. Liver X receptors (LXRs), as the nuclear receptor superfamily, are critical for the regulation of lipid homeostasis. Wang et al. found that PRV infection reduces LXR levels, and activating LXRs suppresses PRV growth while inhibiting them promotes it. LXR activation lowers cellular cholesterol, crucial for PRV entry, and an LXR agonist prevents PRV infection in mice [64]. This suggests that PRV modulates LXR-regulated cholesterol metabolism to aid its proliferation.

Recently, it was discovered that PRV infection elevates the levels of transmembrane protein 41B (TMEM41B), which plays a pivotal role in regulating autophagy and lipid mobilization. Suppressing its expression hinders virus proliferation, whereas its overexpression fosters it. Furthermore, the knockdown of TMEM41B disrupts PRV entry by altering lipid synthesis and affecting the dynamics of clathrin-coated pits, which are essential for virus entry. Interestingly, lipid replenishment reverses these disruptive effects, indicating that TMEM41B governs PRV infection by managing lipid homeostasis [65]. Additionally, Li et al. have uncovered that tryptophanyl-tRNA synthetase 2 (WARS2), a mitochondrial protein vital for protein synthesis, can be upregulated during PRV infection. Biochemical analyses revealed that suppressing WARS2 in PK-15 cells diminishes PRV infection, whereas augmenting it boosts infection. Moreover, decreasing WARS2 levels impairs PRV’s capability to enhance protein and lipid synthesis [66].

## 4. Interaction with Protein Degradation System

The protein degradation system refers to a complex network of biochemical processes responsible for the breakdown and recycling of proteins within a cell. This system plays a crucial role in maintaining cellular homeostasis by regulating the turnover of proteins and eliminating damaged or misfolded proteins [67]. One of the most important protein degradation pathways is the ubiquitin-proteasome system [68]. In this system, proteins destined for degradation are first tagged with ubiquitin molecules, which serve as a signal for the proteasome—a large protein complex that functions as the cellular “protein shredder.” The proteasome recognizes the ubiquitin tags and degrades the tagged proteins into smaller peptides, which can then be further broken down into amino acids and recycled for the synthesis of new proteins [69].

In addition to the ubiquitin-proteasome system, cells also have other proteolytic pathways, such as lysosomal degradation and autophagy, which target different types of proteins or organelles for degradation [70]. Ma et al. found that PRV infection could upregulate the level of low-density lipoprotein receptor (LDLR), which in turn promotes PRV attachment and entry. They further found that LDLR expression is regulated by proprotein convertase subtilisin/kexin type 9 (PCSK9), which could bind to and target LDLR for lysosomal degradation, thus modulating PRV infection [71]. During PRV infection, the host induces autophagy as a defense mechanism against viral replication [72]. This autophagy response is triggered by various cellular stress pathways activated by the virus, such as endoplasmic reticulum (ER) stress. PRV infection leads to the accumulation of unfolded proteins in the ER, causing ER stress and subsequently activating the unfolded protein response (UPR). One of the downstream effects of UPR activation is the induction of autophagy, which aims to restore ER homeostasis by degrading the accumulated unfolded proteins. Xu et.al showed that PRV induced autophagy to promote self-replication via the classical Beclin-1-autophagy-related gene (ATG)7-ATG5 pathway in neurons [73]. Consistently, autophagy downregulation by *platycodon grandiflorus* polysaccharides attenuates PRV replication [74]. Most recently, Chen et al. discovered that the host heat shock protein, DNAJ member B8 (DNAJB8), impairs PRV proliferation by enhancing cellular autophagy. Meanwhile, SRY-box transcription factor 30 (SOX30) controls the expression and activity of DNAJB8 [75]. Sun et.al observed that the inhibition of autophagy could enhance PRV titers. The manipulation of autophagy by PRV is a complex and multifaceted process involving multiple viral proteins and host factors. One such viral protein is the US3 protein kinase, which has been shown to reduce the level of autophagy via the activation of the AKT/mTOR pathways, thereby modulating the autophagic response during PRV infection [76]. The relationship between PRV and autophagy is dynamic and intricate, with the virus exploiting autophagy for its own benefit while the host cell attempts to utilize autophagy as a defense mechanism against viral infection. Further research is warranted to fully elucidate the molecular mechanisms underlying this complex interaction to increase the understanding of PRV pathogenicity.

## 5. Interaction with Apoptosis and Pyroptosis

Apoptosis, a highly regulated form of cell death, plays a pivotal role in maintaining tissue homeostasis, eliminating damaged cells, and defending against viral infections. PRV infection often triggers a complex cascade of events within the host cell, leading to the activation of apoptotic pathways [77]. This process involves both virus-encoded proteins and host cell factors. Specifically, PRV encodes several proteins that can either promote or inhibit apoptosis, depending on the stage of infection and the cellular context. For instance, US3 is known to inhibit apoptosis during the early stages of infection, allowing the virus to replicate efficiently within the cell [78,79]. These proteins can target various anti-apoptotic pathways, such as the inhibition of caspase activation or the upregulation of anti-apoptotic genes. By suppressing apoptosis, PRV ensures a favorable environment for its replication and dissemination [80].

Conversely, other PRV proteins may induce apoptosis during the later stages of infection [81,82]. This can occur through the activation of death receptors on the cell surface or the disruption of mitochondrial function, leading to the release of apoptogenic factors. The induction of apoptosis by PRV may serve as a mechanism to facilitate virus egress or to evade the host immune response by eliminating infected cells. The host cells, in response to PRV infection, also activate apoptotic pathways as a defense mechanism. This involves the upregulation of apoptotic genes, the activation of caspases, and the induction of mitochondrial dysfunction. The host apoptotic response aims to eliminate the infected cells, thereby limiting virus spread and protecting neighboring cells from infection. The interplay between PRV and cell apoptosis highly depends on various factors, including the virus strains, cell types, and host immune status. Understanding the molecular mechanisms underlying this relationship is crucial for developing effective strategies to control PRV infection and its associated pathologies. PRV exhibits a complex relationship with cell apoptosis, manipulating both apoptotic inhibition and induction for its replication and dissemination. The host cell, in turn, mounts an apoptotic response to eliminate infected cells and restrict virus spread. Further studies are warranted to elucidate the precise mechanisms involved in this intricate virus–host interaction.

Pyroptosis, also known as cell inflammatory necrosis, is a form of programmed cell death that involves the continuous swelling of cells until the cell membrane ruptures, releasing cellular contents and triggering a strong inflammatory response [83]. This process is characterized by the activation of inflammatory bodies, which mediate the activation of various caspases [84]. During pyroptosis, a large number of proinflammatory cytokines, such as interleukin-1β (IL-1β) and interleukin-18 (IL-18), are released. They can induce the expression of other factors like tumor necrosis factor-α (TNF-α) and IFN-γ, which help recruit and activate more lymphocytes to the site of infection [85]. The cytokines released during pyroptosis, particularly IL-1β and IL-18, also activate signaling pathways such as nuclear factor-kappa B (NF-κB), p38, and JNK [86,87,88]. This leads to the expression of various other proinflammatory cytokines at the site of infection, further strengthening the immune response. Additionally, these cytokines act as a bridge between the innate and adaptive immune systems, enhancing the overall immune response to viral infections [89]. By inducing cell death through pyroptosis, the host can eliminate virus-infected cells, preventing further viral replication and spread.

Mice infected with the PRV-HLJ strain showed severe symptoms and died within 48–72 h. Cytokine storms, caused by various cytokines like IFN-α, IFN-β, and others, were linked to histopathological changes from PRV. This cytokine secretion pattern indicates a dysregulation of pro-inflammatory cytokines and an imbalance in immune responses. Additionally, PRV activated the pyroptosis pathway, elevating specific protein levels. These discoveries aid in understanding PRV molecular pathogenesis [90]. Zhang et al. showed that PRV infection triggers gasdermin D (GSDMD)-mediated pyroptosis in porcine alveolar macrophage cells, leading to the increased secretion of IL-1β and lactate dehydrogenase (LDH). Caspase-1 activation is involved in this process. Viral replication or protein production is necessary for pyroptotic cell death. PRV activates both NOD-like receptor thermal protein domain associated protein 3 (NLRP3) and IFN-γ inducible protein 16 (IFI16) inflammasomes, linked to reactive oxygen species production and potassium efflux. In PRV-infected tissues, evidence of pyroptosis and inflammasome activation was observed. This research enhances our understanding of PRV-induced inflammatory responses and cell death pathways, aiding in the development of effective treatments for pseudorabies [91].

## 6. Interaction with Other Pathways and Factors

RNA and protein modifications are ubiquitous in cells, regulating various physiological functions [92,93]. PRV is renowned for its ability to manipulate host cell machinery, probably through subtle yet profound modifications of nucleic acids and proteins. PRV has been shown to exploit m6A modification to enhance self-replication [94]. In addition, PRV also manipulates protein post-translational modifications (PTMs) to benefit its own infection. For instance, PRV UL13 induces the phosphorylation of host H2AX, contributing to enhanced viral replication [95]. Recently, succinylation was shown to have regulatory roles in PRV replication, probably via affecting the succinylation of viral proteins [96]. Intriguingly, the global levels of lysine crotonylation in host PK-15 cells were altered and mapped out with PRV infection. Moreover, a large number of PRV proteins, such as IE180, gD, gM, and US2, were discovered to be modified with crotonylation, while their functions are needed to be addressed in the future [97]. The study of PRV interactions with the host, particularly in the context of modifications, offers valuable insights into the molecular mechanisms underlying viral pathogenesis. Future research in this area holds the potential to unveil novel antiviral strategies targeting these critical virus–host interactions.

Neuropeptide S (NPS) and its receptor (NPSR) play crucial roles in various physiological functions within the body, leading to a cascade of intracellular events, including an increase in calcium ion influx and cyclic adenosine monophosphate (cAMP) levels [98]. A recent study has underscored the importance of NPS and NPSR in PRV infection. It was found that the overexpression of NPS could promote PRV infection in mice via elevating the levels of IL-6 mRNA and STAT3 phosphorylation [99]. Heme oxygenase-1 (HO-1) converts heme into biliverdin (BV), iron, and carbon monoxide (CO), which protect cells from various stressors. Zhang et al. explored the effect of HO-1 on PRV replication and the results showed that overexpressing HO-1 inhibited PRV replication, while reducing endogenous HO-1 expression promoted it. Mechanism analysis revealed that HO-1 downstream metabolites, CO and BV/BR, partially mediated its antiviral effect. HO-1 may be a novel endogenous antiviral factor against PRV, and the HO-1/BV/CO system may form a unique antiviral network during PRV infection [100]. Li et al. assessed the activity of Ras homolog family member A (RhoA), a critical cytoskeleton regulator, through chemical inhibitors, gene knockdown, and overexpression to determine its impact on PRV. Inhibition or knockdown of RhoA enhanced PRV proliferation, while RhoA overexpression or activation suppressed it. Additionally, PRV infection disrupted actin stress fibers, and the actin inhibitor cytochalasin D reduced PRV replication, indicating that actin cytoskeleton polymerization aids in PRV replication [101]. Annexin A2 (ANXA2) performs diverse intracellular roles that are tied to numerous viral infections [102]. It was found that ANXA2 interacts with PRV US3 protein and is crucial for PRV replication. PRV or US3 induces ANXA2 translocation and phosphorylation. US3 also binds to Src and enhances its interaction with ANXA2. Inhibitors targeting ANXA2 reduce PRV propagation in vitro and protect mice from PRV infection in vivo. These findings suggest the important role of ANXA2 in alpha herpesvirus pathogenicity and as a potential therapeutic target [103].

## 7. Conclusions and Perspectives

The intricate interaction between PRV and its host cells is a fascinating display of evolutionary adaptation and biological warfare. This review has delved into the nuanced interactions that occur during PRV infection, shedding light on how the virus exploits host machinery for its replication, dissemination, and evasion of immune responses. The ability of PRV to hijack cellular processes underscores its remarkable adaptability and viability. Its manipulation of host signaling pathways, such as those involving autophagy and apoptosis, illustrates how viruses can turn cellular mechanisms meant for maintaining homeostasis against the host itself. The virus’ strategic modulation of the immune system, through both direct and indirect mechanisms, further emphasizes its sophistication in evading host defenses. The study of host–virus interactions in PRV infection not only advances our understanding of virology but also has practical implications for disease control. By elucidating these mechanisms, we can identify potential targets for antiviral therapies and develop more effective vaccines. However, it is important to note that viruses like PRV continually evolve, and thus, our understanding and approach to controlling them must also evolve. In conclusion, the host–virus interaction in PRV infection is a multifaceted and dynamic process that requires continuous investigation.

PRV exerts multiple effects on the host, among which the immunosuppressive effect is more significant. From the interaction mechanisms, PRV could play a role in the modulation of cytokine expression, alter the metabolic pathways of immune cells, and induce the degradation of key immune factors by viral encoded proteins. Nevertheless, future studies could focus on how PRV alters the PTMs of the key immune factors, including cGAS–STING signaling, to impede their functions, thus contributing to self-replication. On the other hand, PRV has been implicated in a spectrum of reproductive abnormalities in both natural and experimental hosts, including infertility, abortion, and fetal death, thus causing huge losses in the breeding industry. PRV employs multifaceted strategies to disrupt normal reproductive function. These mechanisms encompass direct viral cytopathogenicity, the induction of inflammatory responses, and the modulation of hormonal balances. For example, PRV infection may lead to the destruction of reproductive tissues through lytic replication, resulting in impaired ovarian function and reduced fertility. Additionally, the virus triggers robust inflammatory responses, characterized by the infiltration of immune cells and the production of cytokines, which can further disrupt the delicate balance of the reproductive microenvironment. Furthermore, PRV may interfere with the synthesis and metabolism of reproductive hormones, such as gonadotropins and steroids, which are essential for the regulation of reproductive processes. Understanding these interaction mechanisms is crucial for developing effective preventive and therapeutic strategies to mitigate the reproductive consequences of PRV infection and may also provide insights into the pathogenesis of other viruses that affect reproductive health.

The field of host–virus interactions in PRV infection holds vast potential for future research and advancements. As our understanding of these interactions deepens, so do the possibilities for novel therapeutic strategies and preventive measures. First, further elucidation of the molecular mechanisms underlying PRV manipulation of host cell processes promises to reveal new targets for antiviral drugs. By disrupting specific host–virus interaction points, it may be possible to develop treatments that are more targeted and less disruptive to the host’s natural physiology. Second, the development of vaccines that take advantage of our knowledge of virus–host interactions represents a significant opportunity. Such vaccines could potentially offer more targeted and efficient protection against PRV, reducing the burden of disease and preventing outbreaks. Moreover, the study of PRV immune evasion strategies could inform the design of adjuvants that enhance the immunogenicity of vaccines, overcoming the ability of the virus to suppress the host immune response. Additionally, given the evolving nature of viruses, it is crucial to monitor and study emerging PRV strains to identify any changes in their interaction with the host. This proactive approach will enable the research community to stay ahead of potential new challenges posed by the virus. Lastly, with the advent of new technologies, such as CRISPR-Cas9 gene editing, there is an exciting opportunity to create animal models that mimic human disease states more closely. These models could provide invaluable insights into PRV pathogenesis and host response, further accelerating the development of effective treatments and prevention strategies.

In summary, the future of virus–host interaction research in PRV infection is ripe with possibilities for scientific discovery and translational applications. Continued exploration in this field holds the promise of better protecting both animals and humans from the devastating effects of PRV.
